# Successful, Concurrent Cesarean Section and Type A Aortic Dissection Repair in a Patient With Marfan Syndrome and Sickle Cell Trait

**DOI:** 10.1155/cris/6391145

**Published:** 2026-06-18

**Authors:** Emily C. Scheffel, Lauryn A. Ridley, Gabriel A. Ramos, Jack Guiry, Beatriz Da Cruz Alves, Adanna C. Akujuo

**Affiliations:** ^1^ University of Virginia Health Systems, Charlottesville, Virginia, USA, virginia.edu

## Abstract

We present a 23‐year‐old at 35 weeks’ gestation with Marfan syndrome (MFS) and sickle cell trait (SCT) with acute Type A aortic dissection (TAAD) requiring concurrent cesarean delivery and aortic repair. Delivery, hemi‐arch aortic replacement, and coronary reconstruction were successfully performed. Despite intraoperative complexity and postoperative complications, the patient was managed with fewer ischemic complications compared to previous cases of coincident SCT and aortic repair. This case demonstrates critical principles for managing cardiothoracic emergencies in pregnancy with high‐risk conditions. It emphasizes multidisciplinary care and cardiopulmonary bypass (CPB) techniques for optimization of SCT and maternal‐fetal outcomes in aortopathies.

## 1. Introduction

Though rare, Type A aortic dissection (TAAD) during pregnancy is especially complex due to both medical urgency and fetal dependence on maternal vasculature [1, 2]. Marfan syndrome (MFS) increases maternal dissection risk, especially in cases of prior root dilation [3]. Sickle cell trait (SCT) may increase complications and decrease survival in open‐heart surgery and pregnancy. During cardiac surgery, sickling increases due to mechanical stress from cardiopulmonary bypass (CPB), chemical stress from lactic acidosis, increased oxygen requirement, temperature derangements, and inflammatory cascades. Thus, this population is high‐risk for renal failure, stroke, and splenic infarcts [4, 5].

## 2. Case Report

A 23‐year‐old female at 35 weeks’ gestation with MFS and SCT presented with acute onset back, abdominal, and chest pain; emesis; and upper extremity pressure differential. She had a 3‐year history of stable aortic root dilation to 3.6 cm, monitored with yearly CT imaging. Fetal heart rate monitoring on presentation showed no distress. Bedside echocardiogram suggested aortic dissection, prompting chest CT angiography (CTA). CTA revealed acute TAAD from the ascending to the abdominal aorta, involving the right common carotid and brachiocephalic, left subclavian, and proximal vertebral arteries, none of which were occluded (Figure 1, Video 1). The dissection flap extended into the celiac trunk and superior mesenteric artery (SMA) with severe narrowing but good distal reperfusion as far as was able to be evaluated on chest CTA.

**Figure 1:**
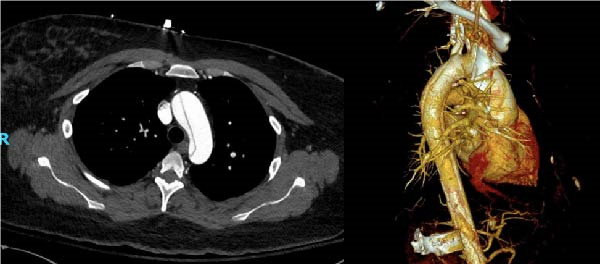
CTA demonstrating Stanford Type A aortic dissection from the tubular to the abdominal aorta, extending superiorly to the right brachiocephalic and common carotid, left subclavian, and proximal vertebral arteries and inferiorly to the celiac and superior mesenteric artery ostia.

Cesarean delivery with subsequent thoracic aortic repair was urgently begun. Findings were confirmed on intraoperative transesophageal echocardiography (Figure 2). Obstetrics successfully delivered the fetus with Apgar scores of 2 and 5 at 1 and 5 min, respectively. Before surgery, the patient’s lactic acid was 1.88 mmol/L.

**Figure 2:**
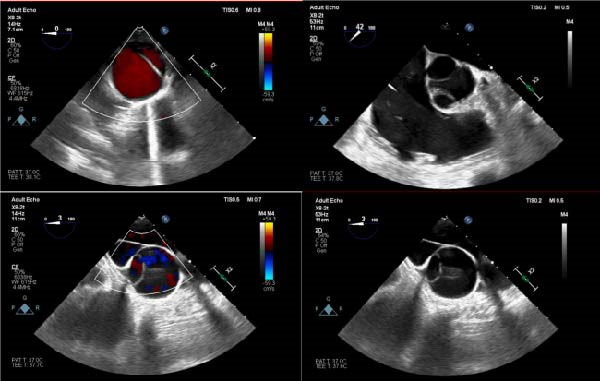
Intraoperative prebypass transesophageal echocardiography demonstrating Stanford Type A dissection.

After fetal delivery, while uterine hemostasis was being achieved, axillary cutdown and graft anastomosis were performed to optimize efficiency. At this point, her lactic acid was noted to have increased to 3.2 mmol/L. The obstetrics team achieved prompt, sufficient hemostasis, pump‐dose heparin was administered, axillary cannulation was performed, and circulatory arrest was initiated. An intimal tear was evident in the ascending aorta with extension just superior to the left coronary ostium. The entry tear was excised, and cryoprecipitate and thrombin were placed between the intima and media.

A graft with a side arm was sewn to the distal portion of the aortic arch, and the side arm was cannulated. A graft was anastomosed to the proximal aortic segment, and the compromised coronary ostium was reconstructed. The proximal segment was then sewn to the distal graft. CPB was weaned without complication. Subsequent hypokalemia and lactic acidosis to 8.88 mmol/L prompted assessment by general surgery for signs of bowel ischemia, which were absent.

Intraoperatively, the lowest hemoglobin was 7.0 g/dL. The patient received eight units of packed red blood cells (pRBCs) as well as fresh frozen plasma, platelets, cryoprecipitate, factor VII, prothrombin complex concentrate, and tranexamic acid. Over the first week, her postoperative course was complicated by hypokalemia, shock liver, coagulopathy, acute hypoxic hypocapnic respiratory failure, lactic acidosis, and RV dysfunction requiring inhaled nitric oxide and epinephrine. Total bypass time was 236 min with a cross‐clamp time of 116 min and 14.15 min of circulatory arrest. The patient was cooled to 18°C for circulatory arrest.

Postoperative CTA showed persistent dissection flap distal to the grafts, terminating at the aortic bifurcation with continued partial occlusion but stable perfusion of the celiac trunk and SMA (Figure 3). Splenic hypoattenuation and perisplenic free fluid were suggestive of evolving splenic infarcts.

**Figure 3:**
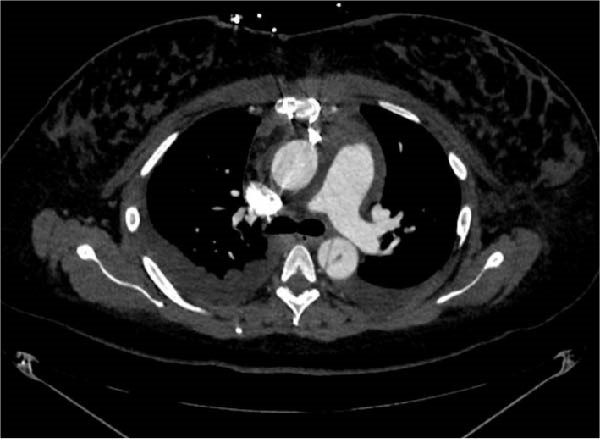
Postoperative CTA demonstrating postsurgical changes of thoracic aortic and proximal hemi‐arch graft repair, without complicating features. Residual dissection of the descending aorta is evident with reported continued improvement of flow in the true lumen.

The patient was discharged on postoperative day 12. Two months later, she presented with idiopathic myocarditis with a left ventricular ejection fraction of 30%. Within months of initiation of goal‐directed medical therapy, her ejection fraction returned to 55%. Imaging soon after discharge demonstrated a 12 cm × 7 cm area of hypoattenuation and fluid collection. More recent imaging has demonstrated splenic lobulation with a stable 3 cm × 7 cm area of hypoattenuation suggestive of infarction versus pseudocyst. Placental pathology demonstrated generalized uteroplacental (vascular) malperfusion and 3.5 cm × 2.5 cm of hemorrhage.

The patient’s residual Type B dissection will continue to be monitored by vascular surgery with imaging every 6 months. Currently, aortic dimensions remain stable at 4.0 cm; however, should further dilation, rapid progression, or unstable perfusion occur, repair will be undertaken.

## 3. Comment

Connective tissue diseases and SCT are known risk factors for pregnancy complications [4]. Previous reports endorse maternal mortality of 20.8% and infantile mortality of 37.5% in the setting of TAAD in pregnant patients with MFS [6]. Despite her extensive dissection, our patient’s prompt presentation and operation aided in minimizing ischemic complications intra‐ and postoperatively compared to prior reported cases of patients with TAAD in the setting of SCT.

Historically, pregnancy‐related changes in flow dynamics, aortic compression by the gravid uterus, and estrogen‐related reductions in vascular elasticity have been implicated as TAAD risk factors [1, 7]. Still, recent studies purport that these associations faced selection bias due to high preadmission mortality in TAAD [3]. Despite controversy over pregnancy being an independent risk factor, pregnant patients with known TAAD predispositions should be considered high risk for vascular complications and provided appropriate surveillance and precautions.

While incidence and treatment of TAAD in MFS are well documented, it remains poorly characterized in patients with SCT. The few reported cases of TAAD in patients with SCT were complicated by splenic infarction and rupture, nonocclusive mesenteric ischemia, coagulopathies, and spinal infarctions. Outcomes were attributed to hypoxia and acidosis induced sickling [8]. Though few similar cases exist, our patient experienced fewer ischemic sequelae than similar SCT cases, which reported splenic rupture or involution, mesenteric ischemia, and long‐term renal and hepatic failure, none of which are present in this case. Despite a complex acute course, our patient had no persistent complications other than her residual Type B dissection.

Consideration of SCT‐related risks prior to CPB is vital. Previous literature has investigated hemodilution and postoperative replacement with pRBCs, preoperative exchange transfusion, and omission of intraoperative suction‐recirculatory devices during CPB for SCT patients [5, 8]. Current guidelines recommend transfusions to maintain <30% HbS in high‐risk operations, a much more easily attained goal in SCT compared to sickle cell disease [8]. In this case, extensive transfusion protocols were activated to increase carrying capacity and decrease HbS in the perioperative periods; however, due to medical urgency, none of the aforementioned steps were attempted. Vigilant intraoperative management of hypoxia and acidosis in our patient aided in mitigating SCT‐related ischemic cascade, demonstrated by limited placental and colonic ischemia and stable, localized areas of splenic infarct. In retrospect, exchange transfusion and limitation of suction‐recirculatory mechanisms may have decreased postoperative coagulopathy.

This case highlights the importance of multidisciplinary care and the management of cardiac surgery patients with SCT and pregnant patients with MFS. In patients with SCT and disease, even in urgent cases, employment of measures to reduce HbS concentration and sickling is vital in preserving remaining splenic tissue and reducing thrombotic events. While our patient, like many TAAD patients, experienced a complex course, we attribute success to multidisciplinary planning and intentional perioperative care.

## Funding

The authors have nothing to report.

## Consent

Consent is obtained.

## Conflicts of Interest

The authors declare no conflicts of interest.

## Supporting Information

Additional supporting information can be found online in the Supporting Information section.

## Supporting information


**Supporting Information** Video 1: Preoperative CT scan demonstrating Stanford Type A aortic dissection from the tubular to the abdominal aorta, extending superiorly to the right brachiocephalic and common carotid, left subclavian, and proximal vertebral arteries and inferiorly to the celiac and superior mesenteric artery ostia, performed at 10 : 21 on the day of the operation.

## Data Availability

Data sharing is not applicable to this article as no datasets were generated or analyzed during the current study.
